# Associations of gestational diabetes and proton density fat fraction of vertebral bone marrow and paraspinal musculature in premenopausal women

**DOI:** 10.3389/fendo.2023.1303126

**Published:** 2024-01-16

**Authors:** Saori Harada, Alexandra S. Gersing, Yannick Stohldreier, Olaf Dietrich, Andreas Lechner, Jochen Seissler, Uta Ferrari, Eleni Pappa, Nina Hesse

**Affiliations:** ^1^ Medizinische Klinik und Poliklinik IV, Diabetes Zentrum - Campus Innenstadt, LMU Klinikum, Ludwig-Maximilians-Universität München, Munich, Germany; ^2^ Institute for Medical Information Processing, Biometry and Epidemiology (IBE), Faculty of Medicine, LMU Munich, Pettenkofer School of Public Health, Munich, Germany; ^3^ Department of Neuroradiology, LMU University Hospital, LMU Munich, Munich, Germany; ^4^ Department of Radiology, LMU University Hospital, LMU Munich, Munich, Germany; ^5^ German Center for Diabetes Research (DZD), Neuherberg, Germany

**Keywords:** bone marrow, spine, paraspinal musculature, gestational diabetes mellitus, magnetic resonance imaging, proton density fat fraction, women in bone research

## Abstract

**Background and objective:**

Fat content in bones and muscles, quantified by magnetic resonance imaging (MRI) as a proton density fat fraction (PDFF) value, is an emerging non-invasive biomarker. PDFF has been proposed to indicate bone and metabolic health among postmenopausal women. Premenopausal women with a history of gestational diabetes (GDM) carry an increased risk of developing type 2 diabetes and an increased risk of fractures. However, no studies have investigated the associations between a history of GDM and PDFF of bone or of paraspinal musculature (PSM), composed of autochthonous muscle (AM) and psoas muscle, which are responsible for moving and stabilizing the spine. This study aims to investigate whether PDFF of vertebral bone marrow and of PSM are associated with a history of GDM in premenopausal women.

**Methods:**

A total of 37 women (mean age 36.3 ± 3.8 years) who were 6 to 15 months postpartum with (n=19) and without (n=18) a history of GDM underwent whole-body 3T MRI, including a chemical shift encoding-based water-fat separation. The PDFF maps were calculated for the vertebral bodies and PSM. The cross-sectional area (CSA) of PSM was obtained. Associations between a history of GDM and PDFF were assessed using multivariable linear and logistic regression models.

**Results:**

The PDFF of the vertebral bodies was significantly higher in women with a history of GDM (GDM group) than in women without (thoracic: median 41.55 (interquartile range 32.21-49.48)% vs. 31.75 (30.03-34.97)%; p=0.02, lumbar: 47.84 (39.19-57.58)% vs. 36.93 (33.36-41.31)%; p=0.02). The results remained significant after adjustment for age and body mass index (BMI) (p=0.01-0.02). The receiver operating characteristic curves showed optimal thoracic and lumbar vertebral PDFF cutoffs at 38.10% and 44.18%, respectively, to differentiate GDM (AUC 0.72 and 0.73, respectively, sensitivity 0.58, specificity 0.89). The PDFF of the AM was significantly higher in the GDM group (12.99 (12.18-15.90)% vs. 10.83 (9.39-14.71)%; p=0.04) without adjustments, while the CSA was similar between the groups (p=0.34).

**Conclusion:**

A history of GDM is significantly associated with a higher PDFF of the vertebral bone marrow, independent of age and BMI. This statistical association between GDM and increased PDFF highlights vertebral bone marrow PDFF as a potential biomarker for the assessment of bone health in premenopausal women at risk of diabetes.

## 1 Introduction

Chemical shift encoding-based water-fat MRI (CSE-MRI), determining the proton density fat fraction (PDFF), is an emerging non-invasive quantification method for bone marrow composition (1–6). In previous studies, bone mineral density (BMD) was inversely correlated with increased vertebral bone marrow fat (7–9). Bone marrow adipocytes are considered insulin-sensitive, by expressing insulin receptors. Under metabolic disturbances, such as obesity and type 2 diabetes (T2D), bone marrow adiposity is induced with impaired bone health (10). The link between elevated bone marrow PDFF and systemic insulin resistance was reported in postmenopausal women with newly diagnosed T2D (11). Systemic insulin resistance is another potential cause of bone fragility via the impairment of osteoblast functions and other pathophysiological mechanisms (12). In postmenopausal women, T2D was associated with an increased fracture risk (13). Paradoxically, patients with T2D often show normal or increased BMD (13). The quantitative computed tomography (QCT)-based assessment of the BMD showed no significant changes within 1 year prior to the occurrence of a vertebral compression fracture (14). On the other hand, a further study demonstrated that over 1 year prior to the occurrence of an incidental vertebral compression fracture, the PDFF had significantly increased in the respective vertebral bodies compared to the PDFF of the vertebral bodies of the controls without vertebral compression fracture (14). Several other studies have indicated that bone marrow PDFF may be predictive for vertebral compression fractures and a potential biomarker for bone health (15, 16).

Furthermore, T2D has been demonstrated to have an impact on other compartments of the body containing fat including the paraspinal musculature (PSM) (17). T2D is known to cause changes in muscle architecture, composed of a shift in myocyte composition, increased myosteatosis (fatty infiltration of skeletal muscle), and a decreased capacity for muscle regeneration (18, 19). These changes are associated with impaired skeletal muscle mass function and degeneration of the skeletal muscles (20) Numerous studies proposed that an intricate cellular and molecular mechanism was responsible, involving insulin, sex hormones, myokines, lipid metabolites, a subset of fibro-adipogenic progenitors, and other factors (18, 20, 21). These pathologic cascades ultimately culminate in increased morbidity and disability (21). Lipid accumulation in muscles of the lower limbs was found to be associated with increased fracture risk in an older population (22). Increased intramyocellular lipids in lower leg muscles, measured with 1H nuclear magnetic resonance spectroscopy, were observed in women with a history of gestational diabetes mellitus (GDM) (23). However, no studies have looked at the associations between a history of GDM and the PDFF of bone marrow or of the PSM.

GDM, a transient disturbance of glucose tolerance, is one of the most common medical complications during pregnancy, with a prevalence of 1.1% to 24.3% (24). Women with a recent history of GDM show characteristics associated with T2D and are at risk of developing T2D (25, 26). A previous study reported an association between a history of GDM and an increased fracture risk (27).

This study aims to investigate whether MRI-based PDFF measurements of vertebral bone marrow and PSM are associated with a history of GDM in premenopausal women.

## 2 Materials and methods

### 2.1 Study participant selection

The study was approved by the local institutional review board (Ethics Commission of the Medical Faculty, Ludwig-Maximilians-Universität München) and all study participants provided written informed consent prior to their participation in the study, which was conducted in accordance with the declaration of Helsinki. Cross-sectional analyses were performed at baseline visits after delivery within a monocentric prospective observational cohort study, as reported previously (25). Women with a history of GDM as well as women following normoglycemic pregnancy (controls) were included in the study, from 6 to 15 months after delivery, between April 2013 and September 2015. The diagnosis of GDM was based on a 75 g oral glucose tolerance test (OGTT) after the 23rd week of gestation following the criteria of the International Association of the Diabetes and Pregnancy Study (IADPSG) recommendations (28). Study participants who underwent MRI after the baseline visit, using the same MRI protocol and MR system, were selected for this study.

### 2.2 Anthropometric data, steps per day, and oral glucose tolerance test

Body weight in kilogram (kg) was assessed using a bioelectrical impedance analysis scale (Tanita BC-418, Tanita Corporation, Tokyo, Japan). For clothing, 0.5 kg was subtracted. Height and waist circumference were measured with an accuracy of 0.5 cm, using a tape measure. BMI was calculated as weight divided by the square of height (kg/m^2^).

As an indicator of daily physical activity, steps per day were tracked among the study participants, using an accelerometer (Aiper Motion 440, v3.2.4.0, Aipermon GmbH). The participants carried the accelerometer for at least 10-14 days except for holidays. The average steps per day were calculated based on the number of days when they were able to carry the device.

A 5-point 75 g OGTT was performed at the baseline visit. Definitions of the American Diabetes Association were used to distinguish between normal vs. pathologic glucose metabolism (impaired fasting glucose 100-125 mg/dl [5.6-6.9 mmol/L]), impaired glucose tolerance (120 minutes of OGTT 140-199 mg/dl [7.8-11.0 mmol/L]), or newly diagnosed T2D (fasting plasma glucose (FPG) ≥ 126 mg/dl [7.0 mmol/L] or 120 minutes of OGTT ≥ 200 mg/dl [11.1 mmol/L])) (29).

For the criteria of metabolic syndrome, we used the International Diabetes Federation (IDF) Worldwide Definition of Metabolic Syndrome for women (1. Waist circumference > 88 cm, 2. Triglycerides ≥ 150 mg/dl, 3. High-density lipoprotein cholesterol < 50 mg/dl, 4. Hypertension as systolic blood pressure ≥ 130 mmHg or diastolic blood pressure ≥ 85 mmHg, 5. FPG ≥ 100 mg/dl) (30). Each required examination was performed at the baseline visit.

### 2.3 Magnetic resonance imaging

MRI scans were scheduled after the baseline visit. Whole-body magnetic resonance examinations were performed with a 3-tesla system (Ingenia, Philips Healthcare, Best, Netherlands) using an anterior body coil and a posterior coil. The latter was integrated into the MR table. Subjects were placed in the scanner in a supine position with arms extended above their head. A slab-selective three-point-echo 3D gradient-echo sequence (Dixon) was used to acquire all echoes in a single TR, using bipolar gradients (repetition time 4.1 ms, first echo time 1.45 ms, second echo time 2.19 ms, third eco 2.93 ms, flip angle 10°, slice thickness 10 mm, gap 0 mm, 400 × 400 matrix, 520×520 mm^2^ field of view). Water and fat images were calculated by the MRI software (Philips Healthcare). The PDFF maps were determined by pixelwise evaluating the ratio of the fat (F) signal over the sum of fat and water (W) signals, F/(F + W) * 100%. The same approach for the fat fraction calculation that we used is described and confirmed to be reproducible in previous literature (31, 32).

### 2.4 Quantitative vertebral body and paraspinal muscle analysis

**Figure 1:**
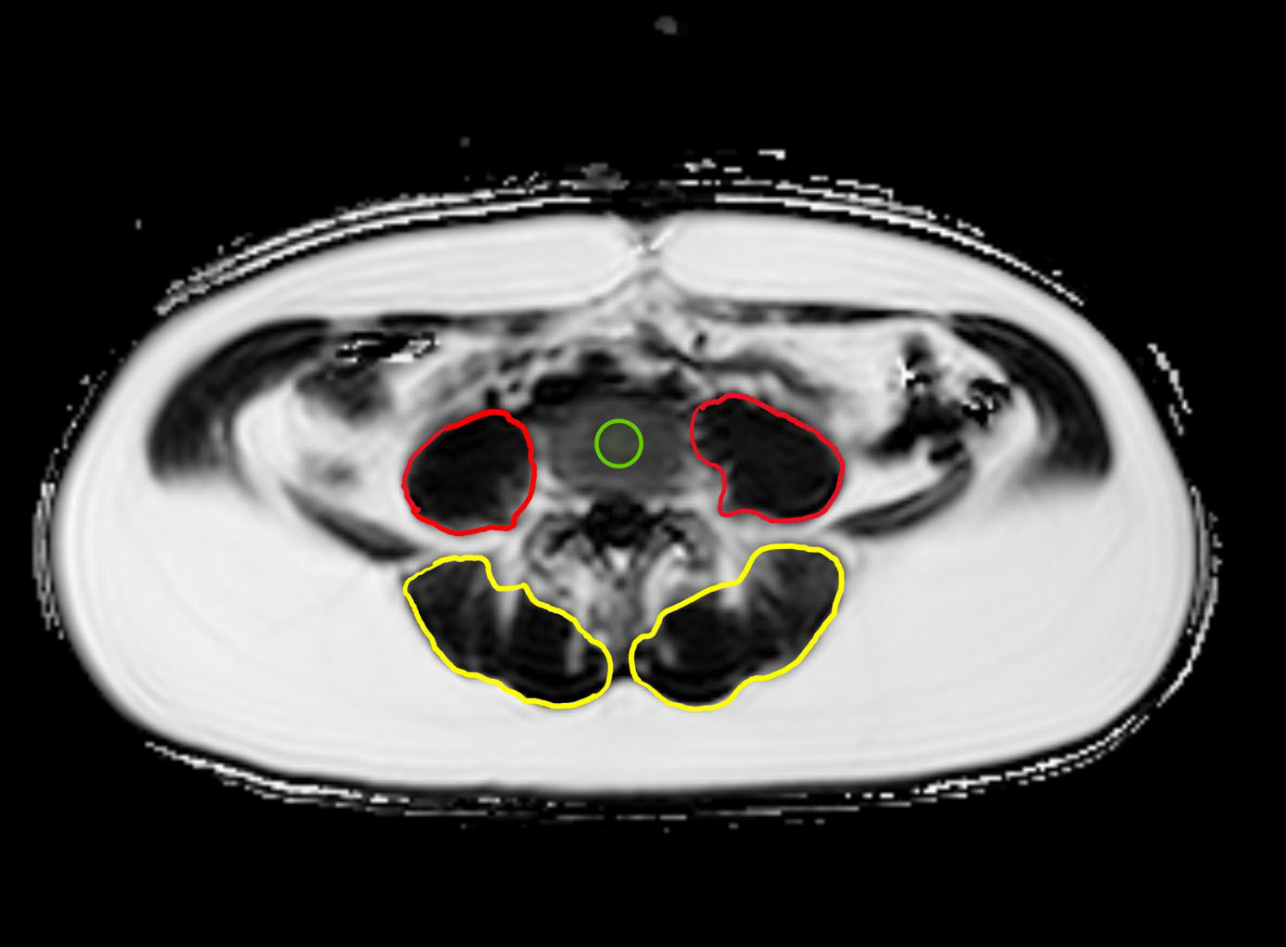
Example PDFF map at the level of L4: Region of interest (ROI) placement in the center of L4 (green) as well as representative segmentations of the autochthonous muscles (AM, yellow) and psoas muscles (PM, red) on both sides.

All MR images were checked for vertebral fractures or vertebral deformities, yet, there were no fractures detected in any of the study participants. Segmentations of the thoracic and lumbar vertebrae and the paraspinal muscles were performed by a trained researcher (Y.S.) and reviewed by two board-certified radiologists (N.H., A.S.G. with 9 and 12 years of experience in musculoskeletal imaging, respectively), primarily to confirm the adequacy of the selected areas of interest excluding other unintended areas such as vertebral discs, on the PDFF maps using Visage PACS (Visage Imaging, Inc., San Diego, CA, United States). The region of interest (ROI) was placed in the center of the vertebral body from Th9 to Th12 and from L1 to L4. The mean value and standard deviation for thoracic or lumbar vertebral bodies were calculated. Beginning at the level of L1, the cross-sectional area (CSA) of the paraspinal musculature (autochthonous muscle (AM) and psoas muscle (PM) on both sides) in cm² was semiautomatically segmented bilaterally on three slices 5 cm apart of the thickest part of the muscle, and then it was averaged. A representative PDFF map with an assessment of CSA and PDFF ROI measurement at the level of L4 is shown in **Figure 1**. All measurements were performed blinded to the clinical data and demographics of the participants. A random sample of 10 subjects was independently analyzed by N.H. after a 6-month interval following the mentioned review process, in order to assess the inter-reader reproducibility. A random sample of 10 subjects was reanalyzed 4 weeks later in order to assess the intra-reader reproducibility.

### 2.5 Statistical analysis

All statistical analyses were performed using RStudio Build 492 “Mountain Hydrangea” (R Foundation for Statistical Computing, Vienna, Austria). The statistical analysis was performed by S.H. (8 years of experience with statistical analysis). All statistical tests performed were two-sided with a level of significance (α) of 0.05. Normally distributed metric variables are expressed as mean ± standard deviation. Non-normally distributed metric variables are reported as median (interquartile range of the first quartile to the third quartile). Pearson correlation was used to assess correlations between normally distributed variables, and Spearman’s rank correlation was used for non-normally distributed variables. To compare groups without adjustments, a two-sample t-test (for normal distributions) and Wilcoxon rank sum test/Mann-Whitney U test (for non-normal distributions) were used for variables with equal variances. Welch t-test (for normal distributions) and Mood’s median test (for non-normal distributions) were used for variables with unequal variances. For categorical variables, the Fisher exact test (if the sample size in one group was less than 5) and the Chi-squared test were conducted. Multivariable linear and logistic regression models were performed to evaluate the associations between measured mean PDFF of the vertebral bodies or PSM and history of GDM, adjusting for age and BMI at the baseline visit. A history of GDM was defined as an independent variable in linear regression models and as a dependent variable in logistic regression models. ROC curves were drawn in order to assess the PDFF cutoff values from the sensitivity and specificity, differentiating between women with and without a history of GDM. The optimal cutoff values were selected to maximize the sum of sensitivity and specificity. The area under the ROC curve (AUC) was computed with a 95% confidence interval. Inter-reader and intra-reader reproducibility for PDFF values were assessed by calculating the intraclass correlation coefficient and the root mean square coefficient of variation (RMSCV) of the differences between the respective measurements.

## 3 Results

### 3.1 Study participant characteristics

A total of 37 women (mean age at delivery was 35.4 ± 3.8 years) with (n=19) and without (n=18) history of GDM were included in this study. No significant differences in age at delivery, in time from delivery to baseline visit, from delivery to MR imaging, and from baseline visit to MR imaging were found between the women with a history of GDM (GDM group) and the women without a history of GDM (control group). The GDM group and the control group did not differ significantly in terms of BMI (GDM group 24.35 (21.14 to 26.92) kg/m^2^ vs. control group 21.91 (20.53 to 25.01) kg/m^2^; p = 0.23).

Out of the 37 women, 13 were categorized as overweight, having a BMI ≥ 25 kg/m^2^ (GDM group, n=8; control group, n=5), and 9 women of the GDM group and none of the control group presented a pathologic glucose metabolism. Out of the 37 individuals, 15 met at least one diagnostic criterion for the metabolic syndrome (GDM group, n=9; control group, n=6) and 2 women of the GDM group fulfilled at least three diagnostic criteria for the metabolic syndrome.

Steps per day were counted among 32 women with (n=17) and without (n=15) history of GDM. Three women showed 10,000 or more steps per day (GDM group, n=1; control group, n=2). The steps per day did not differ significantly between the groups (GDM group 7543 ± 1705 steps vs. control group 7962 ± 1643 steps, p=0.49). Study participant characteristics are displayed in **Table 1**.

**Table 1:** Characteristics of women, differentiated by women with a history of gestational diabetes mellitus (GDM group) and women without a history of GDM (control group).

		GDM group	Control group	p-value
Study participants (n)		19	18	
Age at time of delivery (years)				
	Mean ± SD	36.16 ± 4.07	34.56 ± 3.40	p=0.20 ^a^
	Median (IQR)	36.00 (32.50-39.50)	35.00 (32.00-36.00)	
	Range	29 – 42	28 – 40	
Age at time of baseline visit (years)				
	Mean ± SD	37.11 ± 4.01	35.44 ± 3.57	p=0.19 ^a^
	Median (IQR)	37.00 (33.00-40.50)	36.00 (33.00-37.00)	
	Range	30 – 43	28 – 41	
BMI at time of baseline visit (kg/m^2^)				
	Mean ± SD	25.46 ± 6.45	22.84 ± 3.95	p=0.23 ^b^
	Median (IQR)	24.35 (21.14-26.92)	21.91 (20.53-25.01)	
	Range	18.56 – 44.12	17.47 – 30.56	
Overweight (BMI ≥ 25 kg/m²; n)		8	5	p=0.57 ^c^
Pathologic glucose metabolism (n)		9	0	p ≤ 0.01 ^d^
At least one diagnostic criterion for metabolic syndrome (n)		9	6	p=0.59 ^c^
Three or more diagnostic criteria for metabolic syndrome (n)		2	0	p=0.49 ^d^
Steps per day at time of baseline visit (steps)		(n=17)	(n=15)	
	Mean ± SD	7543 ± 1705	7962 ± 1643	p=0.49 ^a^
	Median (IQR)	7783 (6519-8230)	7811 (7212-8866)	
	Range	4595 – 11200	3682 – 10600	
Time between delivery and baseline visit (months)				
	Mean ± SD	9.46 ± 2.68	9.38 ± 2.04	p=0.82 ^b^
	Median (IQR)	8.77 (7.05-12.17)	9.25 (7.63-11.16)	
	Range	6.13 – 14.53	6.40 – 12.83	
Time between delivery and MRI (months)				
	Mean ± SD	11.05 ± 2.74	11.50 ± 2.05	p=0.57 ^a^
	Median (IQR)	11.27 (9.03-13.03)	12.07 (10.01-13.05)	
	Range	7.23 – 16.60	7.77 – 14.30	
Time between baseline visit and MRI (days)				
	Mean ± SD	47.84 ± 41.84	63.61 ± 40.92	p=0.15 ^b^
	Median (IQR)	33.00 (15.00-74.50)	48.00 (32.25-91.00)	
	Range	5 – 138	16 – 150	

a Two-sample t-test.

b Wilcoxon rank sum test/Mann-Whitney U test.

c Chi-squared test.

d Fisher exact test.

GDM, gestational diabetes mellitus; SD, standard deviation; IQR, interquartile range (the first quartile-the third quartile); BMI, body mass index; MRI, magnetic resonance imaging.

### 3.2 PDFF of the vertebral bone marrow

**Figure 2:**
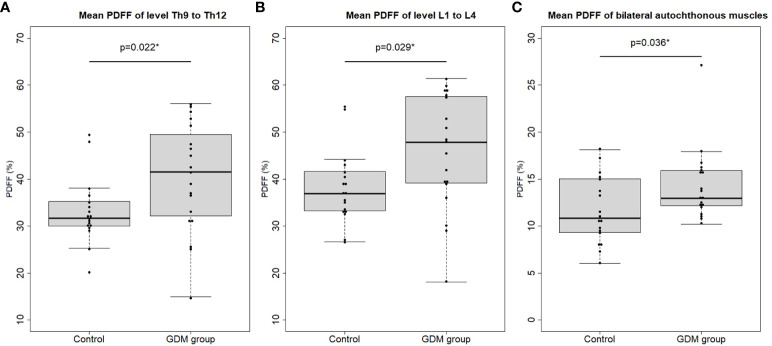
Mean proton density fat fraction (PDFF) of the **(A)** thoracic (level Th9 to Th12) and **(B)** lumbar (level L1 to L4) vertebral bone marrow and **(C)** mean PDFF of the bilateral autochthonous muscles (AM) in the control vs. the women with a history of gestational diabetes mellitus (GDM group). Dots represent the mean PDFF value of each study participant. Asterisks indicate p < 0.05.

By the group comparisons without adjustments, PDFF values of the thoracic (Th9-Th12) and lumbar (L1-L4) vertebral bodies were significantly higher in the GDM group than in the control group (thoracic: 41.55 (32.21 to 49.48)% vs. 31.75 (30.03 to 34.97)%; p=0.02 and lumbar: 45.93 ± 12.22% vs. 38.22 ± 7.79%; p=0.03; **Table 2**, **Figure 2**). After adjusting the analysis for age and BMI using multivariable linear regression analyses, these effects remained significant. History of GDM was significantly associated with the mean PDFF of thoracic vertebral bodies (beta coefficient (β) of history of GDM = 8.94% (95% confidence interval (CI): 2.09 to 15.79%); p=0.01), and with that of lumbar vertebral bodies (β = 9.26% (95% CI 1.93 to 16.59%; p=0.02; **Table 3**).

**Table 2:** PDFF and CSA analyses, differentiated by women with a history of gestational diabetes mellitus (GDM group) and women without a history of GDM (control group).

		GDM group	Control group	p-value
PDFF of thoracic vertebrae from 9 to 12 (percentage)				
	Mean ± SD	40.37 ± 11.60	33.11 ± 7.00	**p=0.022** ^a^
	Median (IQR)	41.55 (32.21-49.48)	31.75 (30.03-34.97)	
	Range	14.95 – 56.09	19.97 – 49.46	
PDFF of lumbar vertebrae from 1 to 4 (percentage)				
	Mean ± SD	45.93 ± 12.22	38.22 ± 7.79	**p=0.029** ^b^
	Median (IQR)	47.84 (39.19-57.58)	36.93 (33.36-41.31)	
	Range	18.11 – 61.47	26.63 – 55.52	
PDFF of the right and left psoas muscles (percentage)				
	Mean ± SD	9.65 ± 2.08	8.31 ± 2.35	p=0.07 ^b^
	Median (IQR)	9.68 (8.06-11.27)	8.29 (6.60-9.92)	
	Range	6.47 – 13.36	4.18 – 13.33	
PDFF of the right and left autochthonous muscles (percentage)				
	Mean ± SD	14.28 ± 3.81	11.67 ± 3.55	**p=0.036** ^c^
	Median (IQR)	12.99 (12.18-15.90)	10.83 (9.39-14.71)	
	Range	10.22 – 27.03	6.03 – 18.11	
CSA of the right and left psoas muscles (cm²)				
	Mean ± SD	7.75 ± 1.70	7.88 ± 0.96	p=0.79 ^d^
	Median (IQR)	7.52 (6.81-8.87)	7.75 (7.21-8.71)	
	Range	4.83 – 11.1	6.33 – 9.45	
CSA of the right and left autochthonous muscles (cm²)				
	Mean ± SD	14.88 ± 3.14	15.70 ± 1.84	p=0.34 ^d^
	Median (IQR)	15.74 (12.68-17.41)	15.24 (14.71-17.02)	
	Range	9.04 – 19.31	12.66 – 18.99	

a Mood’s median test.

b Two-sample t-test.

c Wilcoxon rank sum test/Mann-Whitney U test.

d Welch t-test.

PDFF, proton density fat fraction; CSA, cross-sectional area; GDM, gestational diabetes mellitus; SD, standard deviation; IQR, interquartile range (the first quartile-the third quartile).

The bold values are considered statistically significant.

**Table 3:** Linear regression models, for the association of PDFF or CSA with a history of gestational diabetes mellitus (GDM).

	Unadjusted univariable model		Adjusted multivariable model*	
Dependent variable	βcoefficient of GDM (95% CI of β)	p-value	βcoefficient of GDM (95% CI of β)	p-value
**Mean PDFF of thoracic vertebrae from 9 to 12 (percentage)**	7.26 (0.82, 13.70)	**0.028**	8.94 (2.09, 15.79)	**0.012**
**Mean PDFF of lumbar vertebrae from 1 to 4 (percentage)**	7.71 (0.83, 14.59)	**0.029**	9.26 (1.93, 16.59)	**0.015**
**Mean PDFF of the right and left psoas muscles (percentage)**	1.34 (-0.14, 2.82)	0.07	0.87 (-0.69, 2.43)	0.27
**Mean PDFF of the right and left autochthonous muscles (percentage)**	2.61 (0.15, 5.07)	**0.038**	1.95 (-0.58, 4.49)	0.13
**Mean CSA of the right and left psoas muscles (cm^2^)**	-0.12 (-1.05, 0.81)	0.79	-0.31 (-1.25, 0.63)	0.51
**Mean CSA of the right and left autochthonous muscles (cm^2^)**	-0.82 (-2.55, 0.91)	0.34	-1.66 (-3.31, -0.01)	0.049

*Adjusted multivariable models are adjusted for age and body mass index at baseline visit.

PDFF, proton density fat fraction; CSA, cross-sectional area; GDM, gestational diabetes mellitus; CI, confidence interval.

The bold values are considered statistically significant.

In the multivariable logistic regression analyses adjusted for age and BMI, the odds of having a history of GDM were significantly greater in individuals with higher mean PDFF values of the thoracic or lumbar vertebral bodies (both odds ratios 1.10, 95% CI 1.02 to 1.2: p=0.02; **Table 4**).

**Table 4:** Logistic regression models, for the association of PDFF or CSA with a history of gestational diabetes mellitus (GDM).

:	Unadjusted univariable model		Adjusted multivariable model*	
Independent variable	Odds ratio (OR) for GDM (95% CI of OR)	p-value	Odds ratio (OR) for GDM (95% CI of OR)	p-value
**Mean PDFF of thoracic vertebrae from 9 to 12 (percentage)**	1.08 (1.01, 1.18)	**0.039**	1.10 (1.02, 1.21)	**0.019**
**Mean PDFF of lumbar vertebrae from 1 to 4 (percentage)**	1.08 (1.01, 1.17)	**0.039**	1.10 (1.02, 1.20)	**0.021**
**Mean PDFF of the right and left psoas muscles (percentage)**	1.33 (0.98, 1.89)	0.08	1.22 (0.87, 1.77)	0.25
**Mean PDFF of the right and left autochthonous muscles (percentage)**	1.26 (1.02, 1.63)	0.052	1.21 (0.97, 1.58)	0.13
**Mean CSA of the right and left psoas muscles (cm^2^)**	0.94 (0.57, 1.52)	0.78	0.81 (0.43, 1.43)	0.48
**Mean CSA of the right and left autochthonous muscles (cm^2^)**	0.88 (0.66, 1.14)	0.34	0.72 (0.48, 0.99)	0.07

*Adjusted multivariable models are adjusted for age and body mass index at baseline visit.

PDFF, proton density fat fraction; CSA, cross-sectional area; GDM, gestational diabetes mellitus; CI, confidence interval.

The bold values are considered statistically significant.

**Figure 3:**
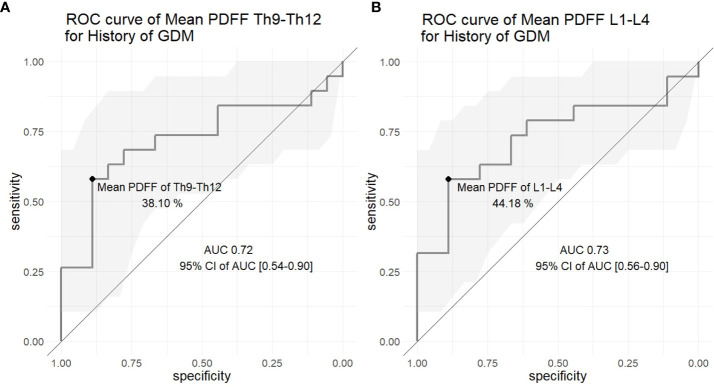
Receiver operating characteristic (ROC) curves of the mean proton density fat fraction (PDFF) of the **(A)** thoracic (level Th9 to Th12) and **(B)** lumbar (level L1 to L4) vertebral bone marrow to differentiate between the control and the women with a history of gestational diabetes mellitus (GDM). The gray area represents the 95% confidence interval (CI) of the area under the curve (AUC).

For the differentiation between women with and without a history of GDM based on the mean PDFF of thoracic and lumbar vertebral bodies, the areas under the ROC curves (AUCs) were 0.72 and 0.73, respectively (**Figure 3**). The optimal thoracic and lumbar vertebral PDFF cutoff values were 38.10% and 44.18%, respectively (sensitivity 0.58 and specificity 0.89 for both).

**Figure 4:**
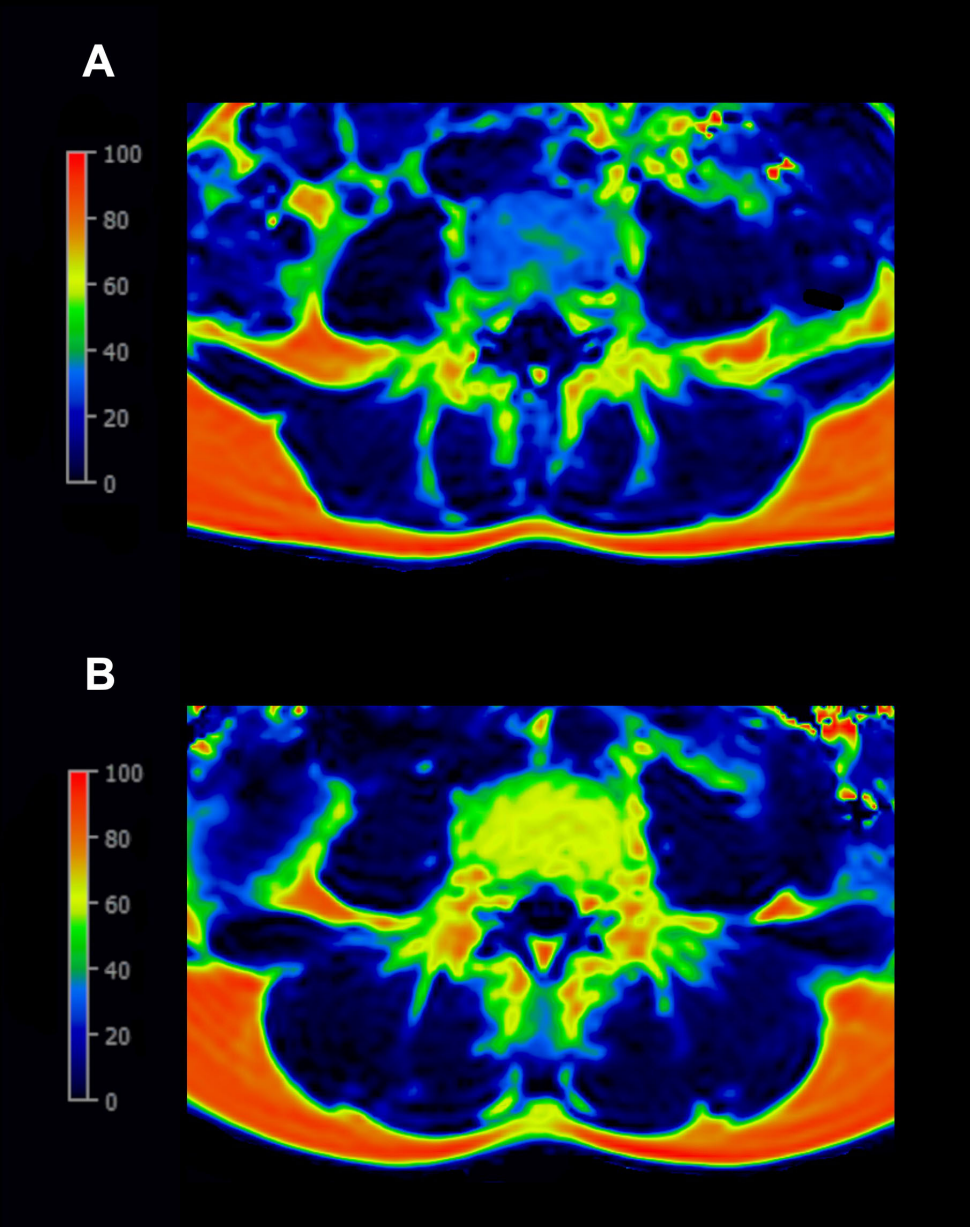
Examples of the color-coded proton-density-fat-fraction (PDFF) map at the level L4: **(A)** A 33-year-old woman after normoglycemic pregnancy (BMI 19.15 kg/m², PDFF of L4 32.13%) with blue indicating lower PDFF values. **(B)** A 37-year-old woman with a history of gestational diabetes mellitus (BMI 18.56 kg/m², PDFF of L4 62.96%) with yellow indicating higher PDFF values.

As examples, the PDFF maps at the level of lumbar (L4) vertebral bone marrow are shown: one in a woman after normoglycemic pregnancy and the other in a woman with a history of GDM (**Figure 4**).

### 3.3 PDFF and CSA of the paraspinal musculature

When analyzing the group comparisons without adjustments, the PDFF values of the autochthonous muscles (AM) were significantly higher in the GDM group than in the control group (12.99 (12.18 to 15.90)% vs. 10.83 (9.39 to 14.71)%; p=0.04; **Table 2**, **Figure 2**). Only in the unadjusted univariable linear regression model, the history of GDM was significantly associated with the mean PDFF value of AM (β = 2.61% (95% CI 0.15 to 5.07%; p=0.04; **Table 3**). No significant differences between the GDM group and the control group were detected in PDFF values of the PM (GDM group 9.65 ± 2.08% vs. control group 8.31 ± 2.35%; p=0.07), in CSA of the AM (GDM group 14.88 ± 3.14 cm² vs. control group 15.70 ± 1.84 cm²; p=0.34) or in CSA of the PM (GDM group 7.75 ± 1.70 cm² vs. control group 7.88 ± 0.96 cm²; p= 0.79). These analyses continued to show no significant associations with the history of GDM after adjusting for age and BMI (p ≥ 0.05; **Tables 3**, **4**).

Neither was there a significant correlation found between CSA and PDFF of the PSM (PM: GDM group r=-0.08, p=0.75; control group r=-0.24, p=0.34; AM: GDM group r=-0.14, p=0.58; control group r=-0.15, p=0.55) nor was there a significant correlation found between the PDFF of the PSM and the PDFF of vertebral bodies (PM and Th9-Th12: GDM group r=0.02, p=0.93; control group r=0.28, p=0.26; PM and L1-L4: GDM group r=-0.02, p=0.93; control group r=0.31, p=0.21; AM and Th9-Th12: GDM group r=-0.27, p=0.26; control group r=0.06, p=0.82; AM and L1-L4: GDM group r=-0.32, p=0.19; control group r=0.10, p=0.70) in any of the groups.

### 3.4 Inter-reader and intra-reader reproducibility

Inter-reader agreement for mean PDFF within the thoracic and lumbar vertebral bodies (Th9 – L4) and the PSM was excellent (ICC, 0.98 [95% CI, 0.96-0.99] and 0.97 [95% CI, 0.96-0.99] for these mean PDFF analyses, respectively).

Inter-reader reproducibility, calculated by the RMSCV, was excellent with < 1.0% (0.95% and 0.97% for these mean PDFF analyses, respectively).

Intra-reader agreement for the corresponding PDFF was excellent (ICC, 0.98 [95% CI, 0.96–0.99] for both mean PDFF analyses).

Intra-reader reproducibility, calculated by the RMSCV, was excellent with < 1.0% (0.91% and 0.93% for these mean PDFF analyses, respectively).

## 4 Discussion

In this study, the vertebral bone PDFF and the paraspinal muscle PDFF and CSA of premenopausal women, with and without a history of gestational diabetes, were investigated. Our study demonstrates that women with a history of GDM (GDM group) show significantly higher PDFF values of the thoracic or lumbar vertebral bodies than women without a history of GDM (control group), independent of age and BMI. Without adjusting for age and BMI, the PDFF of the autochthonous musculature was significantly higher in the GDM group than in the control group, while the CSA was similar between the groups. These statistical findings do not prove any causality in our study.

A previous study reported that the mean lumbar vertebral PDFF was significantly higher in osteoporotic/osteopenic patients than in non-osteoporotic/non-osteopenic patients among an older population (15). A further study identified a significantly higher mean vertebral PDFF increase over 12 months before the occurrence of an incidental vertebral compression fracture compared to the longitudinally measured mean vertebral PDFF in patients without incidental vertebral compression fractures (14). Again, this previous study was performed in an older study population.

Diabetes presents with a wide heterogeneity when looking closely at the diagnosed population (33). This may be the reason for the contradictory results of previous studies regarding marrow fat content in patients with T2D compared to healthy individuals. Some studies showed higher bone marrow fat in healthy individuals (11, 34) or no significant difference in bone marrow fat content between patients with T2D and healthy controls (35–37). One specific diabetic disease subtype is GDM. The diagnosis is being held at lower glucose measures during the oral glucose tolerance test than for T2D during pregnancy. Women with a history of GDM show lower insulin sensitivity and the risk of developing type 2 diabetes is significantly increased compared to women without a history of GDM (25, 26). The pathophysiological pathways in GDM are considered to be less heterogeneous than those in T2D. In terms of bone health, similar to T2D, it has been reported that women with a history of GDM have an increased fracture risk (27). Therefore, in this study we focused on premenopausal women with and without a history of GDM, to investigate PDFF biomarkers in relation to possible early bone changes under the risk of diabetes progression.

Our result is in line with a previous study, reporting that premenopausal women with metabolic syndrome showed significantly higher PDFF values of the lumbar spine than controls (38). In our study cohort, 9 out of 19 women in the GDM group showed a pathologic glucose metabolism, and 2 women in the GDM group fulfilled more than three criteria for the diagnosis of a metabolic syndrome.

Aside from bone marrow fat, metabolic diseases have previously been shown to affect the musculature. A previous study has reported significantly higher PSM PDFF in osteoporotic patients compared to normal controls and found an inverse correlation between paraspinal muscle PDFF and BMD (39). Additionally, higher vertebral PDFF and PSM PDFF were associated with more severe bone fragility (14). In our study, the GDM group showed significantly higher AM PDFF compared to the control group, while there was no significant difference in PS PDFF and PSM CSA between the groups. We found no correlation between AM PDFF and vertebral PDFF in our premenopausal cohort. This result is consistent with a previous study, reporting an association between AM PDFF and vertebral PDFF only in postmenopausal women, but not in premenopausal women (40). It needs to be noted that PSM PDFF in postmenopausal women was significantly higher compared to premenopausal women (40).

Both bone marrow and muscle adiposity have been acknowledged to be associated with physical activity or exercise (41, 42). Several pathophysiological mechanisms are presented, such that physical activity promotes bone marrow fat lipolysis, and that physical inactivity increases intramuscular fat content, while decreasing muscle mass and muscle cross-sectional area (41, 43). We employed steps per day as a measure of daily physical activity. A previous study revealed that young healthy adults could reduce their step count from ~10,000 steps per day to ~1,300 steps per day simply by taking the elevator instead of stairs and by driving instead of walking. Following 21 days of these step reductions, their insulin sensitivity and postprandial lipid metabolism were decreased, and intra-abdominal fat mass increased (44). Our study participants did not show significant discrepancies in steps per day between the GDM group and the control group, however, excluding the impact of physical activity is difficult. We suggest that steps per day can be both the cause and the consequence of the changes in bone and muscle tissue, because the fat-infiltrated bones and muscles can alter the microenvironment, compromising function and performance (21, 22, 45, 46). In this regard, physical activity levels can be influenced both by a history of GDM and by fat infiltrations in bones and muscles, as reflected in higher PDFFs. In this relationship, steps per day would be a collider in the context of directed acyclic graphs (DAG), and adjusting the analysis for this factor may introduce a collider bias. Furthermore, considering our sample size, we decided not to add steps per day as one of the covariates in our linear and logistic regression models.

Our study has several limitations. First, the sample size is limited because only MRI study participants with the identical protocol and system were selected since we prioritized minimizing a potential measurement bias due to different measurement methods. Moreover, given that only those participants who granted consent and were able to complete MRI scans were included in this study, a selection bias cannot be ruled out. Future studies in larger study cohorts are needed to confirm the external validity of our findings. Second, the hormonal status of these women was unidentifiable, which may have had effects on the bone marrow composition. Third, the cohort did not have quantitative information available regarding the BMD (e.g. QCT).

In conclusion, our data suggests that a history of GDM is associated with a higher mean PDFF of the thoracic and lumbar vertebral bone marrow, regardless of age and BMI adjustments, and is associated with a higher mean PDFF of the AM without adjustments in premenopausal women. These findings indicate that PDFF may be a useful biomarker for the assessment of musculoskeletal health in premenopausal women at risk of diabetes. We note that no causality is verified by our findings.

## Data availability statement

The raw data supporting the conclusions of this article will be made available by the authors, upon the further scientific inquiries without undue reservation.

## Ethics statement

The studies involving humans were approved by Ethics Commission of the Medical Faculty, Ludwig-Maximilians-Universität München. The studies were conducted in accordance with the local legislation and institutional requirements. The participants provided their written informed consent to participate in this study.

## Author contributions

SH: Validation, Visualization, Writing – original draft, Writing – review & editing, Data curation, Formal analysis, Investigation, Methodology, Resources, Software, Supervision. ASG: Conceptualization, Data curation, Formal analysis, Funding acquisition, Investigation, Methodology, Project administration, Resources, Software, Supervision, Validation, Visualization, Writing – original draft, Writing – review & editing. YS: Data curation, Formal analysis, Investigation, Methodology, Resources, Software, Validation, Visualization, Writing – original draft, Writing – review & editing. OD: Data curation, Investigation, Methodology, Resources, Software, Supervision, Validation, Visualization, Writing – original draft, Writing – review & editing. AL: Funding acquisition, Resources, Supervision, Writing – review & editing. JS: Conceptualization, Funding acquisition, Investigation, Project administration, Supervision, Writing – original draft, Writing – review & editing. UF: Conceptualization, Investigation, Project administration, Resources, Supervision, Writing – original draft. EP: Resources, Writing – review & editing. NH: Conceptualization, Data curation, Formal analysis, Funding acquisition, Investigation, Project administration, Resources, Software, Supervision, Validation, Visualization, Writing – original draft, Writing – review & editing.
